# The Impact of Weight Loss Secondary to Bariatric Surgery on Telomere Biology: A Narrative Review

**DOI:** 10.3390/biomedicines14020417

**Published:** 2026-02-12

**Authors:** Saleha Khan, Husna Irfan Thalib, Sariya Khan, Yara Osama Aldawood, Dahlia Mirdad, Abdulrahman Alotaibi, Wisam Jamal, Haneen Alnazzawi, Wed Salah, Abeer Zakariyah

**Affiliations:** 1 General Medicine Practice Program, Batterjee Medical College, Jeddah 21442, Saudi Arabia; salehashafi5@gmail.com (S.K.); husnairfan2905@gmail.com (H.I.T.); sariyak2003@gmail.com (S.K.); yaraoad2004@gmail.com (Y.O.A.); 2 Department of Basic Medical Science, Division of Pathology, College of Medicine, University of Jeddah, Jeddah 21589, Saudi Arabia; dsmirdad@uj.edu.sa; 3 Department of Surgery, College of Medicine, University of Jeddah, Jeddah 21589, Saudi Arabia; aalotaibi@uj.edu.sa (A.A.); wjamal@uj.edu.com (W.J.); 4 Department of Surgery, Division of Anesthesiology, College of Medicine, University of Jeddah, Jeddah 21589, Saudi Arabia; htalnazawi@uj.edu.sa; 5 Department of Basic Medical Science, College of Medicine, University of Jeddah, Jeddah 21589, Saudi Arabia; whsalah@uj.edu.sa; 6 Department of Obstetrics and Gynecology, College of Medicine, University of Jeddah, Jeddah 21589, Saudi Arabia; 7 Department of Basic Medical Science, Division of Medical Genetics, College of Medicine, University of Jeddah, Jeddah 21589, Saudi Arabia

**Keywords:** bariatric surgery, weight loss surgery, metabolic surgery, telomerase, obesity, telomere length, oxidative stress, telomere attrition, telomere shortening

## Abstract

The global escalation in chronic obesity and its associated comorbidities has emerged as a substantial public health and economic challenge. This crisis is further compounded by the genomic impact of obesity on telomere length (TL), primarily driven by unchecked oxidative stress. As a result, telomeres shorten, leading to the onset of age-related disorders. In response, effective therapeutic interventions, notably bariatric surgery (BS), have demonstrated significant improvements in patient outcomes by reducing morbidity and mortality rates. Contemporary research has expanded on these benefits, investigating the impact of weight reduction on TL. While the majority of studies support the attenuation of telomeric attrition, ongoing debates surrounding methodological limitations and conflicting results underscore the need for further investigation. This narrative review outlines the current research on the mechanisms that influence telomeres, with a focus on the methodologies used to measure TL. Furthermore, we will examine the most recent findings on the effects of weight loss resulting from surgical intervention on telomere biology. This analysis aims to address specific controversies surrounding this topic and provide evidence-based recommendations valuable to the healthcare sector for mitigating obesity, as well as educating patients about the molecular-level effects of weight reduction resulting from surgical procedures, to enable informed consent.

## 1. Introduction

Obesity is a complex, chronic, noncommunicable disease with significant morbidity and mortality effects [1,2,3,4]. The rapid increase in overweight populations is causing a global health crisis with fatal outcomes and substantial economic impacts. Phelps et al. reported a global doubling of obese adults since 1990, with a fourfold increase in obese children and adolescents, especially in the Middle East and North Africa [5]. Continuing trends could incur annual economic costs of $4.32 trillion [6]. In Saudi Arabia, obesity rates rose from 20% in 2019 to 36% by 2022, according to the Ministry of Health [7,8]. In 2019, obesity accounted for 4.3% of the nation’s annual health expenditures, totaling $3.8 billion, indicating a significant health and economic burden [8].

Given these alarming costs and health implications, comprehensive, cost-effective obesity management is imperative. This typically involves a multidimensional approach that includes both conservative and surgical interventions. Bariatric surgery (BS) is the optimal treatment for individuals who have not succeeded with non-surgical methods [9,10]. It has demonstrated superior efficacy, resulting in substantial weight loss, remission of comorbidities, and improved clinical outcomes [8,9,10,11,12,13,14].

Further research is needed to fully leverage BS’s potential and its long-term benefits by clarifying the relationship between surgical intervention and metabolic adaptations, especially in telomere biology [11]. Telomeres are crucial for genomic stability, preventing chromosomal fusion, recombination, and degradation, thereby regulating cellular lifespan and aging [15,16,17,18]. Diseases like obesity cause early onset of age-related diseases, various cancers, and mortality by excess oxidative stress through telomere attrition [19,20,21,22,23].

Consequently, a substantial body of evidence supports the role of weight reduction in mitigating TL shortening and/or promoting TL elongation secondary to a reduction in inflammation and oxidative stress [17,18,24,25]. Notwithstanding the apparent logic of this relationship and general consensus, controversy arises due to several limitations and contradictory findings that warrant consideration.

A significant limitation is the scarcity of research linking TL to weight loss strategies. Additionally, the methods and instruments used to assess telomere length (TL), primarily employed in correlational studies with cross-sectional designs, offer unique advantages and disadvantages, making it challenging to ensure reliability, robustness, and replicability [19]. Moreover, confounding effects from other weight-loss methods, often recommended in conjunction with surgical interventions, must also be considered [20,21].

These findings suggest that the relationship between weight loss and subsequent alterations in TL is multifaceted. Healthcare professionals are responsible for providing patients with a comprehensive and accurate representation of the benefits and potential adverse effects associated with any medical intervention, including the potential impact of post-BS weight loss on TL.

This review commences with a concise overview of diverse BS techniques. Subsequently, it elucidates the definitions and mechanisms of telomeres and established modulators of TL. The long-term implications of these alterations in telomere biology on patients’ overall health and longevity are evaluated. Finally, the review identifies knowledge gaps in this emerging field of study and proposes directions for future research.

## 2. Materials and Methods

A comprehensive narrative review was conducted to identify previous studies on the association between weight loss secondary to BS and TL. The article selection process encompassed multiple stages, ensuring a comprehensive and contemporary review of the extant literature on the subject matter. The initial stage comprised an extensive literature search through January 2025 to identify relevant studies and publications, using databases such as PubMed, Web of Science, and Google Scholar. Subsequently, combinations of keywords and Medical Subject Headings (MeSH) terms pertinent to the review topic were employed, including ‘Bariatric Surgery’ [MeSH] and ‘Telomere Length’ [MeSH], as well as keywords related to ‘Oxidative Stress’ [MeSH] and ‘Metabolic Health’ [MeSH]. These studies were then systematically screened based on predetermined inclusion and exclusion criteria. For inclusion in the review, articles were required to meet the following criteria: Publication in peer-reviewed journals and written in English. Focus on recent advances in telomere research, specifically studies published within the last 14 years (2010–2024) to ensure data novelty and relevance. Reporting of original research, clinical trials, systematic reviews, meta-analyses, and observational studies. Conversely, studies were excluded if published before 2010 or written in languages other than English.

Ultimately, 11 studies that met the established criteria were included in this review. Initially, two independent junior reviewers and authors assessed the relevance of each study based on its title and abstract. Subsequently, full-text articles of relevant studies were obtained and thoroughly examined to determine their eligibility for inclusion. The reviewers independently extracted data from the selected studies using a standardized form, which included information on study design, participant characteristics, intervention details, and outcome measures. Any discrepancies during the data extraction process were resolved through discussion and consensus among the reviewers and a third reviewer. Reference list screening was also employed to identify relevant articles from the studies that emerged from the initial search. This review specifically focused on identifying the effects, if any, of weight loss secondary to BS surgical interventions on telomere biology.

## 3. Bariatric Surgery (BS)

BS, a significant medical advancement, represents a crucial intervention for individuals with severe obesity and associated health complications [22]. These surgical procedures are designed to modify the digestive system, thereby limiting food intake and nutrient absorption. In addition to facilitating substantial weight loss, BS has demonstrated efficacy in ameliorating, and in some cases resolving, comorbid conditions such as type 2 diabetes, hypertension, and sleep apnea. As with any major surgical intervention, it carries inherent risks and necessitates a lifelong commitment to dietary modifications and medical follow-up to ensure optimal outcomes [23,24]. 

### 3.1. Restrictive Surgeries

These procedures reduce the size of the stomach, thereby limiting the quantity of food an individual can consume, resulting in earlier sensations of satiety (i.e., fullness). Consequently, these procedures can effectively regulate food intake and enhance weight management [22].

#### 3.1.1. Adjustable Gastric Banding (AGB)

This procedure involves placing a silicone band around the upper portion of the stomach to create a small pouch connected to a port located beneath the skin. By adding or removing saline through this port, the band’s tightness can be adjusted. The objective is to regulate the amount of food that passes through the stomach, resulting in a more rapid sense of satiety and reduced hunger [23]. This procedure is typically recommended for individuals with a Body Mass Index (BMI) exceeding 40, or those with a BMI between 35 and 40 who present with obesity-related health complications [26,27].

#### 3.1.2. Laparoscopic Sleeve Gastrectomy (LSG)

This surgical intervention entails the resection of approximately 75–80% of the stomach. The remaining gastric tissue is restructured into a tubular or “sleeve” configuration resembling a banana. This procedure results in a significant reduction in the stomach’s capacity, leading to decreased food intake and subsequent weight loss. Additionally, the excision of the portion of the stomach that produces the hormone ghrelin may contribute to appetite suppression. This procedure is also indicated for individuals with a BMI exceeding 40 or those with a BMI between 35 and 40 who present with obesity-related comorbidities. Consequently, patients typically experience a substantial reduction in excess weight over a period of one to two years, accompanied by improvements in obesity-related health conditions such as type 2 diabetes, hypertension, and obstructive sleep apnea [27].

### 3.2. Malabsorptive Surgeries

These surgical interventions modify the digestive process by reducing the functional length of the small intestine available for nutrient absorption. By restricting food intake and reducing calorie and nutrient absorption, weight loss can be achieved [24].

#### Roux-En-Y Gastric Bypass (RYGB)

This surgical procedure involves creating a small gastric pouch from the upper portion of the stomach, which is subsequently anastomosed to a segment of the small intestine called the “Roux limb”. Consequently, food intake is restricted, and nutrient and energy intake are reduced. Over a period exceeding four decades, this surgical intervention has demonstrated high efficacy in facilitating substantial and sustained weight loss in obese patients. This outcome is particularly notable in individuals with comorbidities such as diabetes, hypertension, sleep apnea, and severe arthritic conditions [28,29].

### 3.3. Combination of Restrictive and Malabsorptive Surgeries

#### Biliopancreatic Diversion with Duodenal Switch (BPD/DS)

This procedure is more complex surgically and involves removing a large portion of the stomach (sleeve gastrectomy) as well as rerouting the intestines (intestinal bypass) to limit calorie and nutrient absorption. This surgery is highly effective for severe obesity, resulting in substantial and lasting weight loss. However, it requires careful monitoring due to the risk of nutritional deficiencies, and patients must commit to lifelong dietary supplements [30,31]. Table 1 below summarizes the various types of BS procedures.

## 4. Telomeres: The Key to Cellular Aging

Telomeres are protective structures located at the termini of chromosomes, comprising repetitive Deoxyribonucleic Acid (DNA) sequences and associated proteins. Their primary function is to maintain chromosomal stability during cellular division, thereby preventing chromosomal damage. With each mitotic event, telomeres undergo progressive shortening, functioning as a biological clock that limits a cell’s replicative potential [32]. Upon reaching a critical length, telomeres induce either cellular senescence, wherein further division is inhibited, or apoptosis. This natural attrition of telomeres is intricately associated with the aging process and the onset of age-related pathologies. Severely shortened telomeres impair cell function and reduce regenerative potential [33]. Telomerase, an enzyme of paramount importance, plays a crucial role in maintaining chromosomal integrity by impeding telomere shortening. Telomerase function depends on several key components. Telomerase Reverse Transcriptase (TERT) serves as the catalytic subunit, adding DNA sequences to telomeres, while Telomerase RNA Component (TERC) functions as the Ribonucleic Acid (RNA) template that guides this process. Furthermore, the shelterin complex, comprising proteins such as TRF1, TRF2, POT1, TIN2, TPP1, and RAP1, binds to telomeres, conferring protection against degradation and regulating telomerase access to these regions [32].

TL is defined as the measure of the number of nucleotide repeats at the terminus of a chromosome. Through successive cell divisions, telomeres undergo attrition and serve as indicators of cellular senescence and replicative history. In the context of obesity, chronic inflammation and oxidative stress resulting from excessive adiposity can accelerate telomere attrition, as these factors contribute to increased cellular turnover and telomere damage. Conversely, the reduction in oxidative stress and inflammation secondary to weight loss that accompanies BS may contribute to telomere stabilization and potentially enhance telomerase activity [33,34]. However, this relationship is complex and influenced by various factors, including the magnitude and sustainability of weight loss, and the patient’s overall health status.

### Modulators of Telomere Length

**Figure 1:**
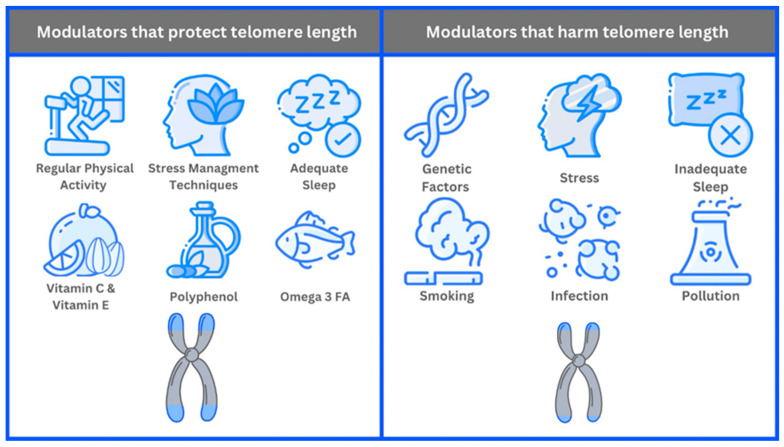
Modulators of TL.

Genetic factors significantly influence baseline TL and the rate of telomere shortening over time. Individuals with longer initial telomeres may experience slower telomere shortening, potentially resulting in a longer cellular lifespan. Lifestyle choices also play a critical role in telomere maintenance [35]. Regular physical activity has been shown to promote longer telomeres, possibly by enhancing antioxidant defenses and reducing inflammation. A balanced diet rich in antioxidants and essential nutrients, such as vitamins C and E, polyphenols, and omega-3 fatty acids, supports cellular health and telomere integrity. These nutrients enhance the body’s ability to combat oxidative stress, a major factor in telomere shortening, by neutralizing harmful free radicals. Stress management techniques, including mindfulness meditation, yoga, and other relaxation practices, can mitigate the negative impact of chronic stress on TL. Chronic stress elevates cortisol levels, accelerating telomere destruction [35,36]. Environmental factors also significantly affect TL and cellular aging processes. For example, exposure to pollution, toxins, and chronic infections can increase oxidative stress and inflammation, thereby accelerating telomere shortening. Pollutants and toxins, such as heavy metals and particulate matter, introduce harmful substances that can directly damage DNA and telomeres. Chronic infections can trigger persistent inflammatory responses, which in turn can lead to telomere attrition [37]. Figure 1 summarizes TL modulators.

## 5. The Link Between Weight Loss Surgery and TL

### 5.1. Studies Reporting an Increase in TL After Weight Loss Surgery

Most included studies reported an increase in TL following BS, although the magnitude, timing, and durability of this effect varied substantially across studies (Table 2). Prospective and cohort investigations demonstrated that TL lengthening was most frequently observed within the first 6–12 months postoperatively, particularly in studies utilizing leukocyte-based measurements assessed by qPCR or flow-FISH methodologies [25,38,39,40,41,42,43,44].

Several studies reported that postoperative TL increases were more pronounced in individuals with shorter baseline telomeres, suggesting a baseline-dependent response and a potential influence of regression toward the mean [20,25,39]. Long-term follow-up data further suggest that sustained weight loss may be associated with gradual telomere stabilization or elongation over extended periods. This effect was most clearly demonstrated in studies with follow-up durations exceeding five years, including the 10-year prospective study by Laimer et al., which reported a significant increase in relative TL following BS compared to population controls [46].

However, telomere lengthening was not uniformly sustained across all time points or cell types. Some studies reported a transient increase in TL during the early postoperative period, followed by partial attenuation at later follow-up, particularly in immune cell subsets such as CD4+ T cells [42]. These findings suggest that early postoperative improvements in TL may reflect short-term changes in immune cell turnover and inflammatory status rather than permanent telomere elongation.

Tissue-specific analyses further highlight heterogeneity in telomere dynamics. Telomeres were consistently longer in visceral adipose tissue compared with leukocytes and subcutaneous adipose tissue, and shorter baseline TL in visceral fat was associated with greater postoperative weight loss, independent of demographic and clinical confounders [39].

Importantly, reported increases in TL, particularly in leukocyte-based measurements, should not be interpreted as unequivocal evidence of true telomere elongation at the single-cell level. Apparent TL lengthening may arise from several biological and technical factors. Leukocyte TL is influenced by immune cell turnover and shifts in the relative proportions of leukocyte subpopulations, each with distinct TL. Changes in inflammatory status and immune activation following BS may therefore alter the average TL measured in peripheral blood without active telomere elongation occurring within the same cells.

In addition, BS is associated with immune profile remodeling, making cell mixture shifts a biologically plausible contributor to observed TL changes in blood-based assays. Methodological considerations further complicate interpretation, as quantitative PCR (qPCR) provides relative TL estimates rather than absolute measurements and is sensitive to DNA quality, amplification efficiency, plate effects, and inter-laboratory variability. In contrast, flow fluorescence in situ hybridization (flow-FISH) allows cell-population–resolved TL assessment and demonstrates greater reproducibility in clinical settings. Consequently, observed post-intervention increases in TL should be interpreted cautiously, particularly when derived from bulk leukocyte qPCR measurements.

### 5.2. Studies Reporting No Change in TL After Weight Loss Surgery

In contrast to the studies mentioned in the section above, some studies found no significant relationship between BS and TL. In a study conducted by Welendorf et al. [39], 48 women were examined before and after six months of the gastric bypass surgical procedure. Several parameters, such as BMI, abdominal circumference (AC), body composition, food intake, and blood samples, were collected for evaluation. DNA and RNA were collected at each moment for TL analysis and gene expression. It was found that there was a reduction in the BMI, AC, total cholesterol, Low-Density Lipoprotein (LD), and triglycerides after the surgery. However, no increase in TL was observed [39].

### 5.3. Sources of Heterogeneity Among Included Studies

Based on the reviewed studies, the results indicate significant heterogeneity, which could contribute to the variability as well as the inconsistencies noted in the TL results following BS. One of the reasons noted is the differing post-surgery follow-up times. These ranged from short-term recovery at 1 to 6 months post-surgery to long-term recovery up to 10 years post-surgery [39,42,43]. Telomere dynamics are indeed influenced by post-surgery time, as short-term changes could result from acute metabolic changes, altered distribution of immune cells, and catabolism [41,45]. The long-term recovery times observed could have more accurately accounted for post-surgery improvements and stress mitigation, as these could have had a more favorable effect on TL maintenance [38,39].

Second, biological sample heterogeneity also contributes to inconsistent findings across studies. While most researchers measured TL in peripheral blood leukocytes, others examined specific immune cell subsets (CD4+ and CD8+ T cells) or distinct adipose tissue compartments (visceral and subcutaneous adipose tissue). TL varies across cell types and tissues due to differences in replicative history and cumulative oxidative exposure, limiting the reproducibility and generalizability of findings across different biological samples. Notably, the variation in the level of TL in the ‘White Cells’ of the body after the operation may not be due to the capacity of the ‘Telomerase’ of the ‘White Cells’ in the body.

Third, methodological variation in TL quantification contributes significantly to heterogeneity. Quantitative PCR protocols, used in most studies, provide only relative TL measurements and are sensitive to DNA quality, amplification efficiency, and inter-laboratory variability [16]. In contrast, flow-FISH, employed in fewer studies, demonstrates superior reproducibility and enables cell-type-specific TL assessment, particularly in immune cell populations. Additional sources of heterogeneity include variation in bariatric surgical procedures, baseline metabolic status (e.g., presence of metabolic syndrome (MetS) or diabetes, degree of postoperative weight loss, and patient demographics such as age and baseline TL [20,40,42]. Collectively, these factors underscore the complexity of interpreting TL changes following BS and highlight the need for cautious synthesis of results.

## 6. Potential Mechanisms Behind the Effects of Weight Loss Surgery on TL

### 6.1. Weight Loss and Metabolic Improvements

The most intuitive change after BS is weight loss. Studies have shown a positive correlation between TL and adiponectin levels, suggesting protective effects against accelerated aging. When weight loss exceeded 16% of the initial weight, plasma free fatty acid (FFA) and C-reactive protein (CRP) concentrations decreased, while plasma adiponectin levels increased significantly [47]. Additionally, weight loss is associated with reduced total, visceral, and pancreatic fat, lower liver lipid levels, and improved insulin sensitivity, all of which are expected to enhance overall glucose metabolism. Elevated blood glucose levels and impaired glucose tolerance can contribute to oxidative stress and glycation, which damage telomeric DNA and shorten telomeres [48]. Enhancing glucose metabolism reduces oxidative stress, thereby maintaining TL [49].

### 6.2. Reduction in Oxidative Stress and Inflammation

**Figure 2:**
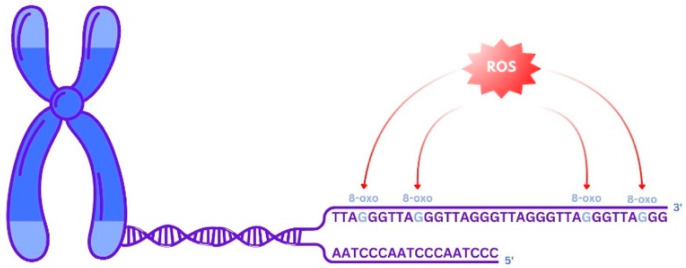
Telomere shortening via oxidative stress targeting guanines, Telomere region’s guanines (G) that are targeted by oxidative stress (blue), causing breakage and damaging telomeric DNA replication, leading to telomere shortening.

Research into the underlying mechanisms proposes that metabolic disturbances associated with obesity, such as insulin resistance and impaired glucose metabolism, induce oxidative stress, thereby contributing to telomere shortening [50]. Lipid accumulation initiates oxidative stress by activating pro-oxidant enzymes and concurrently reducing antioxidant defenses. The telomeric region’s abundance of guanines (G) renders it highly susceptible to oxidative damage, particularly at the GGG triplet, where the formation of 8-dihydro-2′-deoxyguanosine (8-oxodG) occurs, inducing single-strand breaks and impaired replication (Figure 2). This molecule induces pre-mutagenic alterations such as single-strand breaks and impaired telomeric DNA replication, thereby accelerating the rate of telomere attrition. In recent years, BS has proved particularly effective in counteracting the negative health effects associated with morbid obesity. Additionally, the intake of supplements and an antioxidant-rich diet helps combat oxidative stress, thereby reducing its impact on TL [43,51].

Elevated BMI, which is a measure of body fat based on height and weight, promotes the release of inflammatory mediators, including tumor necrosis factor-alpha, interleukin 6, C-reactive protein, and fibrinogen, which are known to suppress telomerase activity [52]. Adipocytes in obese individuals can trigger inflammation by secreting leptin. Leptin and adiponectin are two of the most significant protein hormones produced by adipocytes. The fact that they come from adipocytes and can influence the expression of different markers of systemic inflammation has led to the concept that these proteins are adipocytokines. Researchers discovered strong evidence of a negative relationship between TL and leptin levels in both sexes, regardless of higher BMI or C-reactive protein levels, due to the harmful effects of elevated leptin levels on inflammation, insulin resistance, glucose intolerance, and stress-induced cardiovascular disease [53]. Studies have found that BS effectively reduces chronic low-grade inflammation associated with obesity, which, in turn, leads to TL recovery [26,54].

In the context of the immune response, naive T cells, termed recent thymic emigrants (RTEs), differentiate into memory T cells (CD4+ or CD8+) upon encountering pathogens, enabling robust subsequent immune responses. Alterations in memory T cell subsets—central memory (CM), effector memory (EM), and terminally differentiated EMRA (CD45RA+ EMRA)—are associated with reduced lifespan in severely obese individuals [46]. Chronic systemic inflammation, known as “inflammaging,” accelerates aging and frailty by triggering the release of pro-inflammatory cytokines that activate the immune system, leading to sustained cellular and tissue damage. Aging is characterized by thymic involution, which reduces naive T cell production and increases differentiation into EMRA and CD28-Null T cells, indicating heightened T cell differentiation [46]. These changes, combined with telomere shortening, serve as established markers of human T cell aging and contribute to immune dysregulation in aging and obesity-related metabolic disorders. BS is associated with transient changes in measured leukocyte TL and T-cell differentiation, changes that may primarily reflect shifts in naïve versus memory T-cell populations rather than intrinsic telomere elongation [46].

### 6.3. Changes in Hormonal Milieu

**Figure 3:**
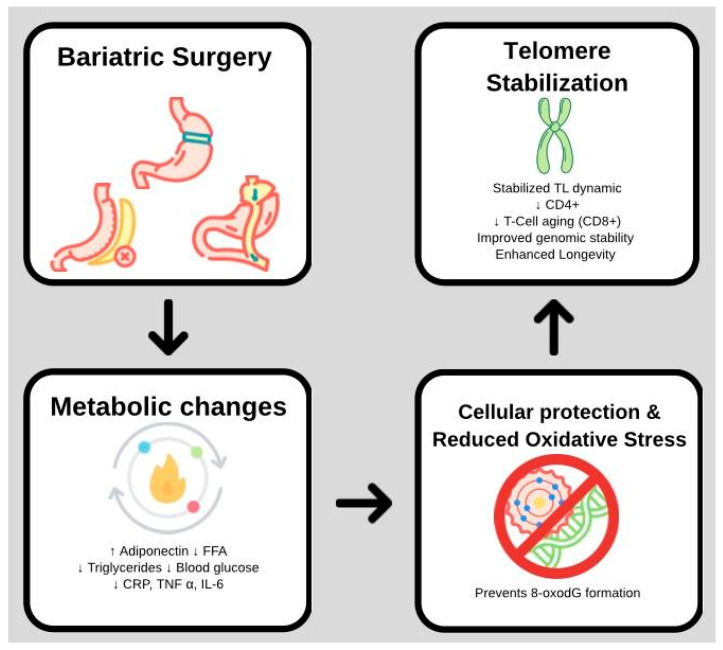
Mechanistic pathway of telomere stabilization post-bariatric surgery. Surgical intervention (LSG/RYGB) triggers metabolic adaptations, including increased adiponectin and reduced systemic inflammation (CRP, IL-6). These changes attenuate oxidative stress at telomeric guanine (GGG) triplets, preventing 8-oxodG formation and DNA breaks. This environment promotes telomere stabilization and genomic stability, leading to improved long-term patient outcomes.

Abnormalities in triglycerides, HDL cholesterol, glucose, blood pressure, and waist circumference are the hallmarks of MetS and dramatically increase health risks [55]. MetS is associated with a persistent systemic inflammatory state in obesity. White adipocyte-produced cytokines, adipocytokines, and adipokines play a crucial role in controlling inflammation and lipid metabolism, promoting insulin resistance, and accelerating telomere shortening [55]. By increasing incretin secretion, restoring islet function, improving peripheral insulin sensitivity to control glucose levels, and lowering metabolic stress that can harm telomeres, BS shows promise in managing diabetes [47] (Figure 3).

Protective factors (anti-inflammatory agents, antioxidants, adiponectin) help maintain TL, while harmful factors (inflammation, ROS, stress) contribute to its shortening.

### 6.4. Hormesis and BS: Adaptive Stress Responses and Telomere Dynamics

From an aging biology perspective, BS may be better conceptualized within the framework of hormesis. Hormesis is a biphasic biological response in which acute or transient stress induces long-term adaptive remodeling that enhances cellular resilience. BS represents a profound metabolic stressor, characterized by abrupt caloric restriction, transient catabolism, redox shifts, immune remodeling, and endocrine reprogramming. These physiological perturbations are not merely reductions in cumulative “damage,” but rather signals that activate stress-response pathways central to metabolic and cellular adaptation.

Within this framework, changes in telomere dynamics following BS may reflect adaptive stress responses rather than linear improvements. Transient telomere shortening or oscillatory changes, often dismissed as methodological variability, may reflect hormetic signatures of immune cell turnover, mitochondrial stress signaling, or stem and progenitor cell redistribution. Over time, these adaptive responses may stabilize telomere maintenance indirectly by reducing chronic inflammatory load and improving metabolic efficiency, rather than through direct telomerase-driven elongation.

## 7. Bariatric Weight Loss Surgery Versus Non-Surgical or Non-Bariatric Surgical Weight Loss Interventions: Which Are More Effective?

The link between TL and BS is complex and still debated. Our review highlights that existing studies present mixed results, underscoring the need for further research. In contrast, numerous studies consistently demonstrate that weight-loss interventions have a positive impact on TL. One study focused on obese adults participating in a 6-month weight-loss program with a bioenteric intragastric balloon (BIB). It found that TL elongation was directly correlated with weight loss, indicating that participants who lost more weight experienced a greater increase in TL [56].

This suggests that weight-loss interventions can not only prevent telomere shortening but also promote telomere elongation. Another study explored the relationship between TL and adipocyte markers in obese adolescents involved in an intensive weight-loss program. It concluded that such interventions significantly increase TL, with longer initial telomeres associated with greater reductions in obesity parameters [57].

A separate five-year study by García-Calzón et al. [58] examined natural weight loss through caloric reduction, diet, and exercise as part of the PREDIMED-NAVARRA trial. The findings indicated a positive relationship between TL and improvements in obesity indices following a Mediterranean diet intervention [58].

Conversely, a pilot study assessing lifestyle changes (Mediterranean diet plus exercise) in severely obese adults found that TL varied widely within this population. Participants with shorter baseline telomeres experienced greater TL and weight loss after a 1-year intervention, along with improvements in mtDNA and total antioxidant capacity, findings consistent with the broader literature [59].

Moreover, two studies have specifically investigated how healthy lifestyle patterns relate to telomere lengthening. Mirabello et al. [60] found a positive correlation between a healthy lifestyle characterized by low-risk factors, high fruit and vegetable intake, lower BMI, and increased physical activity. Similarly, Sun et al. linked a healthy lifestyle, defined by smoking habits, physical activity, body fat, alcohol consumption, and the Alternative Healthy Eating Index, to longer telomeres in women [61].

Finally, a clinical trial examining weight loss in individuals with obesity found that achieving and maintaining a 10% or greater weight loss could lead to TL lengthening, potentially enhancing immune and metabolic function. However, the duration of TL changes beyond 12 months remains unclear and warrants further investigation [62].

Overall, alternative weight-loss interventions appear to have more consistent and positive effects on TL than BS, making this an important consideration for patients weighing their options.

## 8. Discussion

The prevalence of chronic diseases and life expectancy are significantly influenced by obesity. Surgical weight loss interventions, such as BS, are the most effective approach for sustained weight reduction and improvement of obesity-related comorbidities. Surgical weight loss improves patient outcomes by reducing cardiovascular risks [63], decreasing the atherogenic properties of plasma lipoproteins [64], preserving amino acid and protein metabolism, maintaining systemic hormone levels, and enhancing glycemic control [65,66]. However, its effects on TL and stability remain debated [56].

Recent investigations suggest that surgical weight loss may confer benefits for cellular aging and overall health outcomes by impacting TL [67]. Obesity has been consistently shown to negatively affect TL, thereby influencing aging and susceptibility to age-related diseases [68]. A large 2022 cohort study by Schneider et al. [69], including over 450,000 participants, demonstrated that shorter leukocyte TL was associated with moderately increased overall mortality, with elevated organ-specific mortality for respiratory and digestive disorders and hematopoietic neoplasms. Acquired telomere shortening showed susceptibility patterns similar to inherited telomere disorders, and BMI and other lifestyle factors correlated with reduced TL. Limitations included difficulty establishing causation and differentiating genetic versus lifestyle effects [69].

Studies exploring the relationship between BS and TL have yielded mixed results. Ferk et al. [41] examined DNA stability 1 and 6 months post-surgery, measuring TL among other biomarkers. While one assay showed increased TL post-weight loss, the other did not reach statistical significance, highlighting the controversial nature of the BS–TL relationship and the need for extended longitudinal studies [56]. Peña et al. [43] corroborated these findings in a systematic review, emphasizing the paucity and heterogeneity of available studies. Laimer et al. (2016) [46] conducted a 10-year follow-up comparing BS patients with normal-weight controls and observed a relative increase in TL following effective surgical weight loss. However, confounding factors were noted, and larger studies with extended follow-up were recommended [46]. Similarly, Chandru et al. [42] reported a significant increase in TL at 6 and 12 months post-BS in 16 South Indian patients; however, the small sample size, short follow-up, and reliance on serum samples were limitations. Hohensinner et al. [45] found statistically significant increases in TL 24 months after BS, correlating with weight loss and reduced telomere oxidation.

Jongbloed et al. [40] observed a transient increase in T-cell TL post-laparoscopic Roux-en-Y gastric bypass, followed by shortening at 12 months. The study highlighted the ambiguous relationship between obesity, MetS, and TL in circulating leukocytes and T cells, as well as the potential influence of CD28null T-cell populations. Dersham et al. [20] reported TL lengthening in participants with the shortest baseline TL after 3–5 years post-surgery, likely mediated by improved diet, increased physical activity, and reduced oxidative stress. Formichi et al. found that relative TL was shorter in obese subjects than in age-matched controls and decreased further in the immediate postoperative period, likely due to the catabolic state [70]. Regression to the mean may also contribute to apparent increases in TL in longitudinal studies and should be considered when interpreting modest changes. Peña (2020) further confirmed that obesity severity negatively impacts TL, with the most pronounced differences at six months post-surgery, though the effect diminished by 24 months [43].

In contrast, non-surgical weight-loss interventions appear to have more consistent, favorable effects on TL. Studies of intermittent fasting, including Ramadan fasting, have demonstrated improvements in metabolic parameters, reductions in oxidative stress, and stabilization or modest elongation of TL. Ramadan intermittent fasting, for example, has been associated with weight loss, improved insulin sensitivity, and reductions in inflammatory markers in obese and MAFLD/MASLD patients, which may indirectly support TL maintenance [71,72]. These findings suggest that structured, non-surgical interventions may complement, or in some cases exceed, the benefits of surgical approaches for TL preservation. A key biological mechanism potentially underlying the effects of both surgical and non-surgical weight loss on TL is autophagy. Autophagy is a highly conserved and essential cellular process through which cells preserve homeostasis and adapt to stress by degrading and recycling intracellular components [73].

BS and intermittent fasting are known to upregulate autophagy, thereby reducing oxidative stress, removing damaged cellular components, and promoting cellular homeostasis. Enhanced autophagic activity may protect telomeres from oxidative damage, contributing to telomere stabilization or elongation. This mechanism provides a plausible link between weight loss, reduced oxidative stress, and telomere preservation, offering a conceptual framework for interpreting heterogeneous findings in BS studies [74,75].

Although TL is frequently discussed as a marker of biological aging, it should not be interpreted as a direct or causal determinant of longevity. Rather, TL represents a dynamic and context-dependent biomarker that reflects cumulative cellular replication, immune cell turnover, metabolic state, and inflammatory burden. Consequently, changes in TL observed following BS should be interpreted cautiously, as apparent stabilization or elongation does not necessarily indicate cellular rejuvenation or reversal of aging processes. Instead, telomere dynamics likely reflect downstream adaptive responses to shifts in metabolic and inflammatory signaling rather than serving as a primary driver of improved health span. Another important consideration in longitudinal telomere studies is the potential influence of regression to the mean. Individuals with shorter baseline TL may appear to exhibit telomere lengthening on follow-up due to statistical regression and measurement variability rather than true biological change. This is particularly relevant when observed differences are small relative to assay noise, as is often the case with qPCR based TL measurements. Moreover, epigenetic remodeling may represent an upstream regulatory mechanism governing telomere biology during weight loss and metabolic surgery. BS has been shown to induce widespread epigenetic reprogramming linked to insulin sensitivity, inflammatory signaling, and mitochondrial metabolism. It is therefore plausible that observed changes in TL are secondary manifestations of broader epigenetic adaptations rather than primary drivers of improved cellular aging. Future studies integrating epigenomic profiling with telomere dynamics will be essential to clarify causality and identify dominant regulatory mechanisms underlying post-surgical metabolic resilience.

Overall, evidence indicates that BS can increase TL in certain populations, particularly in long-term follow-up and in individuals with short baseline TL. However, the early postoperative period may involve catabolic stress, oxidative stress, and methodological variability in TL assessment, contributing to inconsistent findings. Non-surgical interventions, especially intermittent fasting, may exert more consistent benefits through metabolic improvements and autophagy upregulation. Future studies should incorporate standardized TL measurement techniques, account for baseline TL distribution, and include extended follow-up to better define the sustained effects of surgical and non-surgical weight loss interventions.

## 9. Clinical Implications and Future Directions

The relationship between surgical weight loss and TL is significant, especially in the context of biological aging and metabolic disorders. The outcomes of these weight-loss surgeries significantly affect metabolic parameters, including lipid profiles, glucose metabolism, and inflammatory markers, which are linked to telomere biology. A positive influence of TL indicates that these interventions can confer benefits beyond weight loss, such as anti-aging effects. Several studies have shown that shorter TL is associated with aging-related diseases, such as diabetes and cardiovascular disease [76,77].

While this review provides an overview of the impact of surgical weight loss on TL, it is important to acknowledge some limitations. First, the conflicting findings across studies may be attributed to differences in study design, measurement techniques, and patient demographics. The inconsistency prevents us from drawing a definitive conclusion about the role of surgical interventions in telomere dynamics. In addition, interpretation of results is further complicated by small sample sizes or the absence of long-term follow-up. Therefore, addressing these limitations through larger-scale, more robust studies is crucial to advancing our understanding of this matter. The focus should be on longitudinal studies to track changes in TL before and after the intervention, with particular emphasis on diverse populations to account for demographic variations. Mechanistic studies exploring how surgical weight loss affects telomere dynamics via pathways such as oxidative stress, inflammation, and metabolic changes are also needed. Interdisciplinary collaboration between surgeons, geneticists, and molecular biologists will be essential to fully understand these processes. Moreover, incorporating TL as an outcome measure in clinical trials could help clarify whether telomere changes are directly due to surgical intervention or secondary to weight loss and metabolic improvements. Such insights may not only enhance the personalization of surgical interventions but also pave the way for the development of non-surgical therapies that can promote telomere maintenance and overall longevity in a similar manner. Finally, the role of epigenetics in shaping this relationship is crucial to elucidate, as epigenetics orchestrates telomere biology. The process involves DNA methylation and histone modifications that regulate TL and its stability, with significant implications for cellular aging and disease.

## 10. Conclusions

BS is an effective intervention for reducing obesity related morbidity and mortality and is associated with changes in molecular markers of cellular aging, including TL. Current evidence indicates that telomere responses to surgical weight loss are heterogeneous and time-dependent rather than uniformly progressive. Early postoperative telomere lengthening reported in several studies likely reflects an adaptive response to metabolic and inflammatory stress, with subsequent stabilization as systemic regulation improves.

Within a hormesis framework, BS may function as a controlled metabolic stressor that induces transient catabolic and immune remodeling, followed by improved cellular homeostasis through reductions in oxidative stress, chronic inflammation, and metabolic burden. Accordingly, observed fluctuations in TL are more consistent with adaptive recalibration than with sustained elongation.

Telomere stabilization or modest elongation following BS should therefore be interpreted as one component of a broader adaptive response involving metabolic, immunologic, and epigenetic pathways, rather than as a direct surrogate for longevity. Future studies incorporating longer follow-up, cell-resolved and tissue-specific telomere measurements, and integration of genomic and epigenomic factors are needed to clarify the clinical significance of telomere dynamics after surgical weight loss and to inform personalized therapeutic strategies.

## Figures and Tables

**Table 1:** Summary of the BS procedures.

Category	Procedure	Description
Restrictive surgeries	Adjustable Gastric Banding (AGB)	Involves placing a silicone band around the upper part of the stomach to create a small pouch. The band is adjustable, allowing control over food intake by making the patient feel full more quickly [22,23].
	Laparoscopic Sleeve Gastrectomy (LSG)	A procedure where 75–80% of the stomach is removed, leaving a tube-shaped “sleeve.” This reduces the stomach’s capacity, leading to decreased food intake. It also removes the part of the stomach that produces the hunger hormone ghrelin [27].
Malabsorptive surgeries	Roux-En-Y Gastric Bypass (RYGB)	A procedure that reduces stomach size and bypasses a portion of the small intestine, limiting food intake and nutrient absorption. It has been effective for decades in helping obese patients achieve significant and sustainable weight loss [24].
Combination of Restrictive and Malabsorptive Surgeries	Biliopancreatic Diversion with Duodenal Switch (BPD/DS)	It is a complex surgery that combines both restrictive and malabsorptive techniques. It involves removing a large part of the stomach and rerouting the intestines, significantly reducing calories and nutrient absorption. Requires lifelong dietary management [28,29].

**Table 2:** Summary of the 11 Studies investigating TL changes after BS.

Publication	Study Design	Country	Patient Sample Size and Type	Methods of TL Measurement	Results and Conclusion
Ferk et al., 2023 [41]	Clinical Trial	Austria	BS patients (*n* = 35) Blood samples collected one day before the operation and 1 month and 6 months after the surgery	qRT-PCR	TL increased after 6 months of surgery.
Rolles et al., 2023 [25]	Cohort Study	Germany	BS patients (*n* = 45) Baseline TL in lymphocytes and granulocytes was measured 11 ± 3.3 days (mean ± SEM) before surgery	flowFISH	Post-BS, TL increased in lymphocytes and granulocytes, correlating with weight loss over an average of 5.5 months suggesting beneficial effects on biomarkers of aging beyond weight reduction.
Ospanov et al., 2021 [38]	Single-centre, prospective, three-arm RCT	Kazakhstan	BS patients (*n* = 60) Change in leukocyte TL in all BS treatment groups was measured at 12 months after baseline	qRT-PCR for TL measurement using the single-copy gene sequence ratio method (Cawthon technique).	Two different surgical groups showed significant increases in TL: LOAGB-OSPAN increased by 2.02 units, LMGB-OAGB by 2.07 units, whereas HDER increased by 0.28 units.
Welendorf et al., 2021 [39]	Prospective Study	Brazil	BS patients (*n* = 48) Anthropometric, body composition, and food intake data, as well as venous blood samples (for biochemical indicators, TL and gene expression analysis), were collected at each moment.	qRT-PCR	No significant change in TL
Chandru et al., 2021 [42]	Prospective Study	India	BS patients (*n* = 16) TL, mtDNAcn, serum adiponectin, glycated hemoglobin and high- sensitivity C-reactive protein levels were analysed before surgery and at 6 and 12 months post-surgery	PCR	TL significantly increased at 6 months and persisted 12 months postoperatively compared to baseline.
Gurung et al., 2020 [44]	Longitudinal cohort study	Singapore	BS patients (*n* = 91) Pre-surgery TL was measured in leukocytes, subcutaneous adipose tissue, and visceral adipose tissue of 91 patients undergoing BS. Linear regression in 70 patients analysed the link between pre-surgery TL and weight loss percentage at 6 or 12 months.	qRT-PCR	Telomeres were longer in VAT than in leukocytes and SAT. Individuals in the lowest VAT TL tertile experienced greater weight loss, independent of age, sex, ethnicity, surgery type, diabetes, preoperative BMI, and follow-up duration.
Peña et al., 2020 [43]	Cohort Study	Spain	BS patients (*n* = 94) All patients were evaluated before surgery and during the postoperative period (t6m, t12m, and t24m) for body mass index and metabolic variables	qRT-PCR and telomere sequence to single-copy gene sequence ratio method.	Patients with class III obesity showed significantly shorter TL at baseline than those patients with class II obesity.
Jongbloed et al., 2019 [40]	Non-randomized prospective cohort study	Netherlands	BS patients (*n* = 107) The TL of CD4+ and CD8+ T cells were determined.	flowFISH	A significant increase in CD4+ TL was seen after 3 and 6 months postoperatively. However, at month 12, a decrease in RTL was recorded.
Hohensinner et al., 2018 [45]	Cohort Study	Austria	BS patients (*n* = 58) Whole blood samples for DNA isolation were collected before surgery and at 24 months, aliquoted and frozen immediately.	qRT-PCR	Increase in TL after surgery
Dersham et al., 2017 [20]	Cohort Study	United States	BS patients (*n* = 50) Selected patients included those with readily available DNA from blood collected before surgery and DNA from blood collected between 3 and 5 years after surgery.	q-PCR	Sixty percent of the individuals in the study observed an increase in TL.
Laimer et al., 2016 [46]	Prospective Study	Austria	BS patients (*n* = 142) 110 participants from each study were matched by age and sex to compare changes in TL	q-PCR	TL increased significantly by 0.024 ± 0.14 in all patients within 10 years after surgery.

qRT-PCR: Quantitative real-time reverse-transcription PCR, PCR: Polymerase chain reaction, q-PCR: Quantitative polymerase chain reaction, flowFISH: flow fluorescent in situ hybridization, RTL: relative telomere length.

## Data Availability

No new data were created or analyzed in this study.

## Abbreviations

The following abbreviations are used in this manuscript:

TL	Telomere Length
BS	Bariatric Surgery
BMI	Body Mass Index
DNA	Deoxyribonucleic Acid
RNA	Ribonucleic Acid
TERT	Telomerase Reverse Transcriptase
TERC	Telomerase RNA Component
TRF1	Telomeric Repeat Binding Factor 1
TRF2	Telomeric Repeat Binding Factor 2
POT1	Protection of Telomeres 1
TIN2	TRF1-Interacting Nuclear Factor 2
TPP1	TINT1/PTOP/PIP1 Protein 1
RAP1	Repressor/Activator Protein 1
AGB	Adjustable Gastric Banding
LSG	Laparoscopic Sleeve Gastrectomy
RYGB	Roux-en-Y Gastric Bypass
BPD/DS	Biliopancreatic Diversion with Duodenal Switch
VAT	Visceral Adipose Tissue
SAT	Subcutaneous Adipose Tissue
WBC	White Blood Cells
qPCR	Quantitative Polymerase Chain Reaction
qRT-PCR	Quantitative Real-Time Polymerase Chain Reaction
PCR	Polymerase Chain Reaction
flowFISH	Flow Fluorescence in Situ Hybridization
RCT	Randomized Controlled Trial
MetS	Metabolic Syndrome
LOAGB-OSPAN	Laparoscopic One Anastomosis Gastric Bypass—Ospan Modification (unstapled pouch and connection)
LMGB-OAGB	Laparoscopic Mini-Gastric Bypass/One Anastomosis Gastric Bypass
RTL	Relative Telomere Length
CD4+/CD8+ T cells	Cluster of Differentiation 4 and 8 Positive T Lymphocytes
CD31	Cluster of Differentiation 31 (marker of thymic output)
mtDNAcn	Mitochondrial DNA Copy Number
LDL	Low-Density Lipoprotein
HDL	High-Density Lipoprotein
ROS	Reactive Oxygen Species
